# Acute limb ischemia with concomitant splenic and renal infarcts: Thromboembolic events revealing COVID-19

**DOI:** 10.1016/j.amsu.2021.102646

**Published:** 2021-07-29

**Authors:** Samia Berrichi, Zakaria Bouayed, Sara Berrajaa, Sanae EL. Mezzeoui, Amal Moujahid, Siham Nasri, Houssam Bkiyar, Imane Skiker, Brahim Housni

**Affiliations:** a Anesthesiology and Intensive Care Unit Department, Faculty of Medicine and Pharmacy Oujda, Mohammed VI University Hospital Center, Mohammed University 1st, Oujda, Morocco; b Radiology Department, Faculty of Medicine and Pharmacy Oujda, Mohammed VI University Hospital Center, Mohammed University 1st, Oujda, Morocco; c Oujda Medical Simulation Training Center, Faculty of Medicine and Pharmacy, Oujda, Morocco

**Keywords:** COVID-19, Thromboembolic, Limb ischemia

## Abstract

**Introduction:**

Since December 2019, the coronavirus 19 (COVID-19) pandemic continues to spread worldwide and has caused millions of deaths. Although the main manifestation of the disease is acute respiratory distress syndrome, hypercoagulability resulting from hypoxemia leads to venous thromboembolism and arterial thrombosis, which have a fatal prognosis for COVID-19.

**Case report management:**

We report the case of patient admitted to our unit for management of a concomitant limb ischemia, splenic and renal infarcts associated to a COVID-19 infection with favorable outcomes after thrombectomy and therapeutic doses of heparin.

**Conclusion:**

Systemic procoagulant manifestations related to a COVID-19 infection is a newly described phenomenon responsible of cardiovascular and neurological disorders.

## Highlights

• COVID-19 is a complex and multi-phased disease. The occurrence of arterial and venous thromboembolic events has been described throughout literature and linked to poor prognosis.
• Our case report demonstrates the likelihood of developing an arterial thrombosis even in the absence of cardiovascular comorbidities.
• Thromboembolic involvement during COVID-19 should be considered, and a deeper understanding of the pathophysiology is needed for optimal and management.

## 1 Introduction

COVID-19 Disease has become the main concern in the medical community since the worldwide spread of the virus.

Caused by a coronavirus, named SARS-CoV-2, which is mainly transmitted by droplets or by direct contact with an infected person, this infection can have multiorgan involvement with different clinical expressions [1,2].

Even if the pulmonary infection is the main clinical presentation, other complication patterns, especially cardiovascular, may be revealing of the disease and can be fatal [2].

We report a case of a patient with acute limb ischemia, splenic and renal infarcts revealing a COVID-19 infection.

## 2 Case description

A 45 years old male, with no medical history other than active smoking, presented to the Emergency Room for acute abdominal pain located in the left upper part with no other symptoms. Otherwise, our patient reported an occasional cough over the last 4 days.

The clinical examination finds a conscious patient hemodynamically stable with low oxygen saturation of 70 % on room air, physical examination revealed paleness of the right leg as well as pain and pulselessness suggesting acute limb ischemia.

A nasopharyngeal swab was performed to look for the presence of SARS-CoV-2 and a total-body CT angiography was performed to investigate the cause of the acute abdominal pain and limb ischemia.

**Fig. 1:**
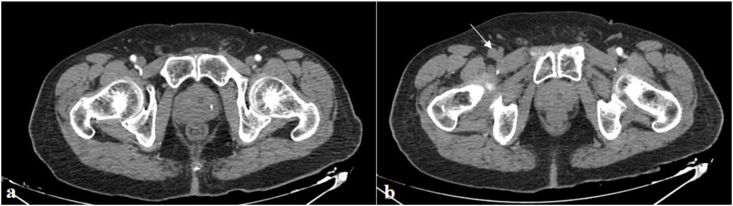
Axial C + Arterial Phase a: symmetrical enhancement of both common femoral arteries b: Occluded right common femoral artery.

**Fig. 2:**
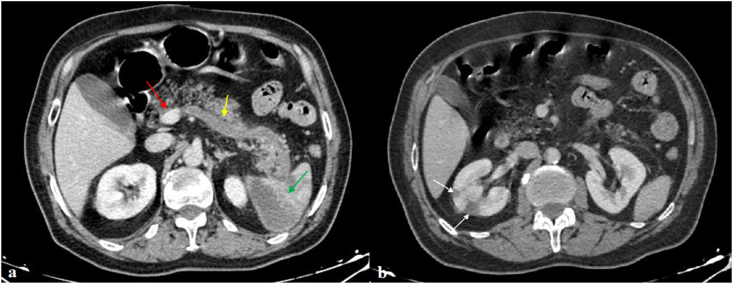
a: Axial C+ of the abdomen in portal venous phase. Red: enhancement of the portal veinYellow splenic vein thrombusGreen triangular hypo enhancing lesion in the spleen compatible with splenic infraction b: Wedge shaped hypodensity of the right kidney consistent with renal infraction. (For interpretation of the references to colour in this figure legend, the reader is referred to the Web version of this article.)

**Fig. 3:**
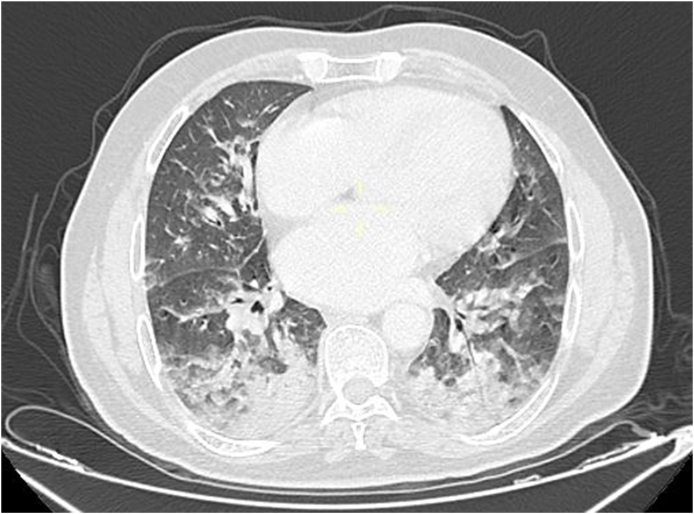
Axial lung window showing multiple ground-glass opacities and lower lobe consolidative areas.

The CT angiography showed a total occlusion of the right common femoral artery (Fig. 1), splenic and renal infarcts and thrombosis of the splenic vein (Fig. 2). Lung CT scan confirmed an interstitial COVID-19 pneumonia (Fig. 3). The EKG was normal and transthoracic echocardiography didn't show any cardiac thrombosis.

**Fig. 4:**
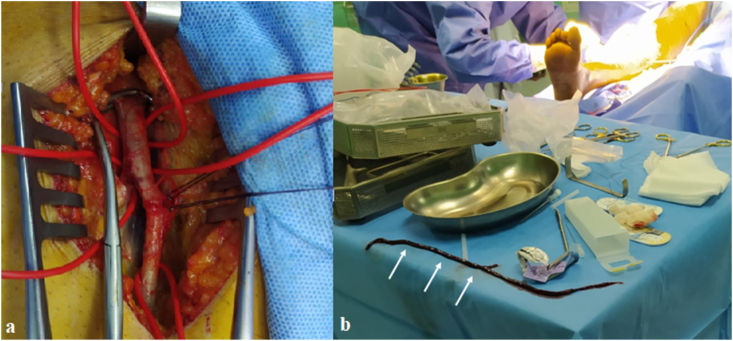
a: Common femoral artery thrombo-embolectomy using a Fogarty catheter b: preoperative image showing the extracted thrombus from the right lower limb. The clot was approximately 30cm long.

The patient admitted to the operating room, he received an 80 units/kg IV induction dose of Unfractionated Heparin, and a Fogarty thrombectomy was performed. Over 30 cm sized thrombus was extracted (Fig. 4) The patient was then transferred to ICU for the management of his pneumonia. He was also tested positive for COVID-19.

The patient was started on a continuous IV infusion of 18 units/kg/hr of unfractionated heparin and the evolution was favorable with complete recovery of the lower limb and the disappearance of his abdominal pain.

Additional treatments included rehydration using Saline, Paracetamol, Azythromycine, Dexamethasone, Vitamins C and D, Zinc and optimization of electrolyte balance.

He was gradually weaned from oxygen and was discharged from ICU after 10 days.

## 3 Discussion

Today, COVID-19 is a pathology with a high mortality rate and the main cause remains respiratory failure due to coronavirus-induced lung damage, which is responsible for severe ARDS [3].

However, cardiovascular complications can be significantly disrupted and exacerbated by SARS CoV-2 infection, even in patients without associated co-morbidities [4].

The incidence of thromboembolic events in SARS-CoV-2 infection is common and has been widely reported in the literature [1,2,5] including large-vessel ischemic stroke and acute upper or lower limb ischemia and more recently, abdominal visceral infarctions including renal and splenic infarctions had been reported [6,7].

In a prospective Dutch cohort of 184 patients with COVID-19 pneumonia, the occurrence of an arterial or venous thromboembolic event was observed in 31 % of cases with predominantly venous manifestations (25 Pulmonary embolism, 1 deep venous thrombosis, 2 catheter thromboses) and only 3 arterial events (ischemic stroke) [2].

Various physiopathological mechanisms resulting in the COVID-19 related hypercoagulability state were identified, mostly immunological and inflammatory components such as the complement cascade activation [8], the macrophage activation syndrome [9], the antiphospholipid antibody syndrome [10], as well as the cytokine storm [11]. Also, an overreaction of the Renin angiotensin system was incriminated [12]. Hypoxemia is also directly involved in hypercoagulability by synthetizing ‘Hypoxia Inducible Factors' [2].

All these mechanisms, mainly target the vascular endothelium resulting in a disturbance of its anticoagulant, antithrombotic and profibrinolytic properties which are prominent, and over stimulate its procoagulant potential which is physiologically minor. This procoagulant potential involves an abnormal activity of tissue factor which amplifies significantly factors VII and X enzymatic capacity resulting in thrombin generation and clot formation. The endothelial cell also releases pre-stored von Willebrand factor (vWf) enabling platelet aggregation and thrombus assembly. In parallel the dysfunctional endothelium manufactures plasminogen activator inhibitor-1 (PAI-1) which antagonizes the endogenous fibrinolytic properties of the endothelial surface [13].

This whole physiopathological pattern falls into the hypothesis of COVID-19 being an endothelial disease [13], highlighting the role of the SARS-Cov-2-induced endotheliitis in creating a hyper coagulant state rendering COVID-19 patients more susceptible to all sorts of thromboembolic events.

The treatment and prevention of thrombotic events in COVID- 19 are still controversial. The use of prophylactic-dose low-molecular-weight heparin (LMWH) has been shown to be associated with lower mortality in patients with severe COVID-19 or D-dimer levels more than 6 times the upper normal limit [6].

The Guidelines recommend the use of standard prophylactic dose anticoagulants in all hospitalized patients with COVID-19 in the absence of a clear contraindication. The routine use of intermediate- or full-therapeutic doses of anticoagulation is not strongly supported by current guidelines [6].

Our patient had simultaneous acute limb ischemia, kidney and spleen infarction. Although the patient had no respiratory symptoms on admission, a chest CT scan revealed typical COVID-19 pneumonia.

The absence of lower limb pain in the presence of acute limb ischemia is dependent on the simultaneous presence of intense abdominal pain, due to a spleen infarction, which distracts attention from leg pain.

Systemic inflammation and a hyper coagulant state due to SARS CoV-2 infection with severe pneumonia are probably responsible for the arterial thrombosis that occurred in our patient [14], even in the absence of cardiovascular co-morbidities.

It is also likely that the onset of limb ischemia, amplified the coagulation cascade leading to thrombosis.

## 4 Conclusion

COVID-19 is a complex disease, involving viral, inflammatory and thrombotic phases. It generally affects the pulmonary system with respiratory distress syndrome, but a systemic procoagulant state may develop, leading to atypical presentations such as cardiovascular and neurological disorders.

Therefor a better understanding of the pathophysiology is essential for optimal and rapid patient management.

Informed consent was obtained from the patient prior to submitting this case.

This work has been reported in line with the SCARE 2020 Guidelines [15].

## Please state any conflicts of interest

The authors state that they have no conflicts of interest for this report.

## Ethical approval

The ethical committee approval was not required given the article type (case report).

## Please state any sources of funding for your research

This research did not receive any specific grant from funding agencies in the public, commercial, or not-for-profit sectors.

## Consent

Written informed consent was obtained from the patient for publication of this case report and accompanying images.

## Author contribution

Berrichi Samia: Study conception, Data collection; data analysis; writing review & editing. Bouayed Zakaria: Study conception, data analysis. Berrajaa Sara: Contributor. Bkiyar Houssam: Supervision and data validation. Housni Brahim: supervision and data validation.

## Registration of research studies

As this manuscript was a case report with no new medical device nor surgical techniques, not prior registration is required.

## Guarantor

BERRICHI SAMIA.

BOUAYED M ZAKARIA.
